# Diagnostic Indications and Endoscopic Correlates of Fecal Occult Blood Testing in Hospitalized Patients

**DOI:** 10.3390/diagnostics16182921

**Published:** 2026-09-10

**Authors:** Ferney Samuel Contento-Anaya, Fabio Andres Contento-Anaya, Mario Montoya Jaramillo, Fernando Barros Gomez, Daniel Botero Alonso, Mileidys Correa Monterrosa, Alex Dominguez-Vargas, Henry J. González-Torres

**Affiliations:** 1 Programa de Especialización en Medicina Interna, Universidad del Sinú, Cartagena 130001, Colombia; contec0809@hotmail.com (F.S.C.-A.); montoyaj7@hotmail.com (M.M.J.); epidemiologoposgrados1@unisinucartagena.edu.co (M.C.M.); 2 Departamento de Gastroenterología, Clínica Santa María, Sincelejo 700001, Colombia; fabiocont2@hotmail.com (F.A.C.-A.); fernando.goamez@gmail.com (F.B.G.); 3 Centro de Investigaciones en Ciencias de la Vida, Facultad de Ciencias de la Salud, Universidad Simón Bolívar, Barranquilla 080001, Colombia; daniel.botero@unisimon.edu.co; 4 Data Analysis and Mining Department, Data & Project Consulting Service, Barranquilla 080001, Colombia; aadominguez17@hotmail.com

**Keywords:** occult blood, gastrointestinal hemorrhage, anemia, colonoscopy, endoscopy, gastrointestinal

## Abstract

**Background:** FOBT is widely used for colorectal cancer screening in asymptomatic populations but is frequently applied in hospitalized patients with anemia or suspected gastrointestinal bleeding despite limited supporting evidence. **Objectives:** To evaluate the clinical utility of fecal occult blood testing (FOBT) in hospitalized patients, focusing on its indications and its association with endoscopic findings while accounting for differential endoscopic verification. **Methods:** We conducted a retrospective study of 160 hospitalized adults who underwent FOBT at a medium-complexity referral center. Demographic, clinical, and endoscopic data were extracted from electronic medical records. Group comparisons used Wilcoxon rank-sum, chi-square, or Fisher’s exact tests; multivariable logistic regression, verification-bias-corrected diagnostic accuracy (Begg–Greenes method), and multiple imputation assessed the association between FOBT and endoscopic findings. **Results:** FOBT was positive in 61.3% of patients. Anemia was the main indication (51%), followed by melena (26%) and hematochezia (6.9%). FOBT positivity was significantly associated with anemia at admission (82% vs. 68%, *p* = 0.044) and hematochezia (10% vs. 0%, *p* = 0.007). Endoscopic verification differed markedly by the FOBT result (71% vs. 29%), and no significant association between FOBT positivity and abnormal endoscopic findings persisted after multivariable adjustment or bias correction, for either EGD (OR 2.01, 95%CI 0.66–6.10) or colonoscopy (OR 4.58, 95%CI 0.39–54.3). **Conclusions:** FOBT shows no robust diagnostic association with endoscopic pathology, upper or lower, once differential verification is accounted for. Its main actionable finding was frequent guideline-discordant use in patients with overt bleeding, identifying institutional stewardship as the primary target for improving diagnostic efficiency in inpatient care.

## 1. Introduction

Fecal occult blood testing (FOBT) has been widely used as a non-invasive tool for colorectal cancer screening in asymptomatic populations, demonstrating effectiveness in reducing colorectal cancer-related mortality when implemented within organized screening programs [1,2]. However, its use has progressively extended beyond this context and is now frequently applied in hospitalized patients as part of the diagnostic evaluation for anemia or suspected gastrointestinal bleeding [3].

In the inpatient setting, gastrointestinal bleeding—either overt or occult—is a common and clinically relevant condition, particularly among older adults and patients with multiple comorbidities [4]. Anemia is a frequent reason for hospital admission and often triggers extensive diagnostic evaluation, including laboratory testing and endoscopic procedures. In this context, FOBT is frequently used as an initial and readily available test to detect gastrointestinal blood loss, despite limited evidence supporting its diagnostic utility in hospitalized or symptomatic patients [5,6,7].

Importantly, this widespread use of FOBT in hospitalized patients often contradicts current clinical guidelines. International recommendations consistently discourage its use in patients with overt gastrointestinal bleeding, such as melena or hematochezia, as these scenarios warrant direct endoscopic evaluation without prior fecal testing [8,9,10]. Nevertheless, FOBT continues to be used in these settings, reflecting a persistent gap between guideline-based recommendations and real-world clinical practice.

Furthermore, the diagnostic performance of FOBT differs substantially between asymptomatic screening populations and hospitalized patients. In the latter, higher rates of false-positive results may occur due to comorbidities, medication use, dietary factors, or active mucosal disease, limiting its specificity and clinical interpretability [3,7]. In addition, prior studies have suggested that FOBT has limited sensitivity for detecting upper gastrointestinal bleeding—partly due to hemoglobin degradation during intestinal transit—while its positivity may correlate more strongly with lower gastrointestinal sources, particularly colonic pathology [11,12].

Despite these limitations, FOBT remains widely used in inpatient practice, raising concerns regarding its clinical utility, cost-effectiveness, and potential to delay definitive diagnostic procedures. This is particularly relevant in low- and middle-income countries, including Colombia, where inefficient use of diagnostic resources may have a disproportionate impact on healthcare systems and patient outcomes [13]. In such settings, inappropriate test utilization may contribute to unnecessary healthcare costs and delays in the diagnosis of clinically significant gastrointestinal disease.

Although previous studies have evaluated the performance of FOBT in selected populations, data on its real-world use and diagnostic implications in hospitalized patients—particularly in Latin American settings—remain limited, and few have accounted for differential endoscopic verification between FOBT-positive and FOBT-negative patients, a factor that can distort diagnostic association estimates [14,15]. This gap hinders the development of rational, evidence-based diagnostic strategies in inpatient care.

Therefore, this study aimed to evaluate the clinical utility of fecal occult blood testing—understood as its diagnostic association with endoscopic gastrointestinal findings [16]—in hospitalized patients at a medium-complexity healthcare institution in Colombia. Specifically, we sought to characterize the clinical indications for FOBT ordering, determine the proportion of positive and negative results, assess the association between FOBT results and subsequent endoscopic findings while accounting for differential endoscopic verification, and evaluate the extent to which institutional use of FOBT aligns with current clinical guideline recommendations.

## 2. Materials and Methods

### 2.1. Study Design

We conducted an observational, retrospective, and analytical study in hospitalized adult patients at a medium-complexity referral center in Colombia. No interventions were performed, and all diagnostic and therapeutic decisions were made by the treating physicians according to standard institutional practice.

### 2.2. Study Population

The study population consisted of adult patients hospitalized during the study period who underwent fecal occult blood testing as part of their diagnostic evaluation. Patients were identified through institutional electronic medical records. All consecutive eligible cases with available clinical data and FOBT results were included, regardless of whether subsequent endoscopic evaluation was performed, as this decision was made independently by the treating physician.

### 2.3. Variables

The main study variable was the fecal occult blood test (FOBT) result, categorized as positive or negative. Demographic variables included age and sex. Clinical variables included indication for FOBT request (anemia, melena, hematochezia, and others), admission diagnosis, comorbidities, medication use (antiplatelet or anticoagulant therapy), and relevant gastrointestinal history.

Diagnostic outcome variables included endoscopic findings from esophagogastroduodenoscopy (EGD) and colonoscopy, classified as normal or abnormal, and specific pathological findings such as ulcers, diverticulosis, hemorrhoids, polyps, and suspected neoplasia. Institutional utilization variables included requesting department and type of diagnostic procedure performed after FOBT testing. As not all patients underwent EGD or colonoscopy, endoscopic result variables and specific pathological findings were defined, and their denominators calculated, only among patients who underwent the corresponding procedure. The FOBT technique (guaiac-based or immunochemical) was determined by clinical and laboratory availability and was not uniformly recorded across patients.

### 2.4. Statistical Analysis

Data normality was assessed using the Lilliefors-corrected Kolmogorov–Smirnov test. As quantitative variables showed non-normal distributions, they were summarized as median and interquartile range (IQR). Categorical variables were described using absolute and relative frequencies.

Comparisons of quantitative variables between groups (by sex and by FOBT result) were performed using the Wilcoxon rank-sum test (Mann–Whitney U test). For categorical variables, Pearson’s chi-square test or Fisher’s exact test was applied when at least one expected cell count was less than 5. For statistically significant binary comparisons, unadjusted odds ratios (ORs) with 95% confidence intervals were calculated as effect-size measures. When a zero cell was present, the Haldane–Anscombe continuity correction was applied.

Multivariable logistic regression was used to identify independent predictors of abnormal endoscopic findings, reporting odds ratios with 95% confidence intervals: FOBT result, age, and antiplatelet/anticoagulant use for EGD, and FOBT result and age for colonoscopy (restricted given the limited number of verified cases). Sensitivity, specificity, positive and negative predictive values (PPV and NPV), positive and negative likelihood ratios (LR+ and LR−), and overall diagnostic accuracy were calculated among verified patients. Ninety-five percent confidence intervals for naive estimates were calculated using the Wilson score method for proportions and the log-normal method for likelihood ratios. These measures were corrected for differential verification using the method of Begg and Greenes [17]. Ninety-five percent confidence intervals for the verification-bias-corrected estimates were obtained by bootstrap resampling with 3000 replicates. As a sensitivity analysis, multiple imputation (50 imputations, Rubin’s rules [18]) re-estimated these associations assuming non-verification depended only on observed covariates (FOBT result, age, sex, symptoms, and comorbidity). Statistical significance was defined as a two-sided *p*-value < 0.05. All analyses were performed using R statistical software (version 4.5.0) [19].

## 3. Results

**Figure 1:**
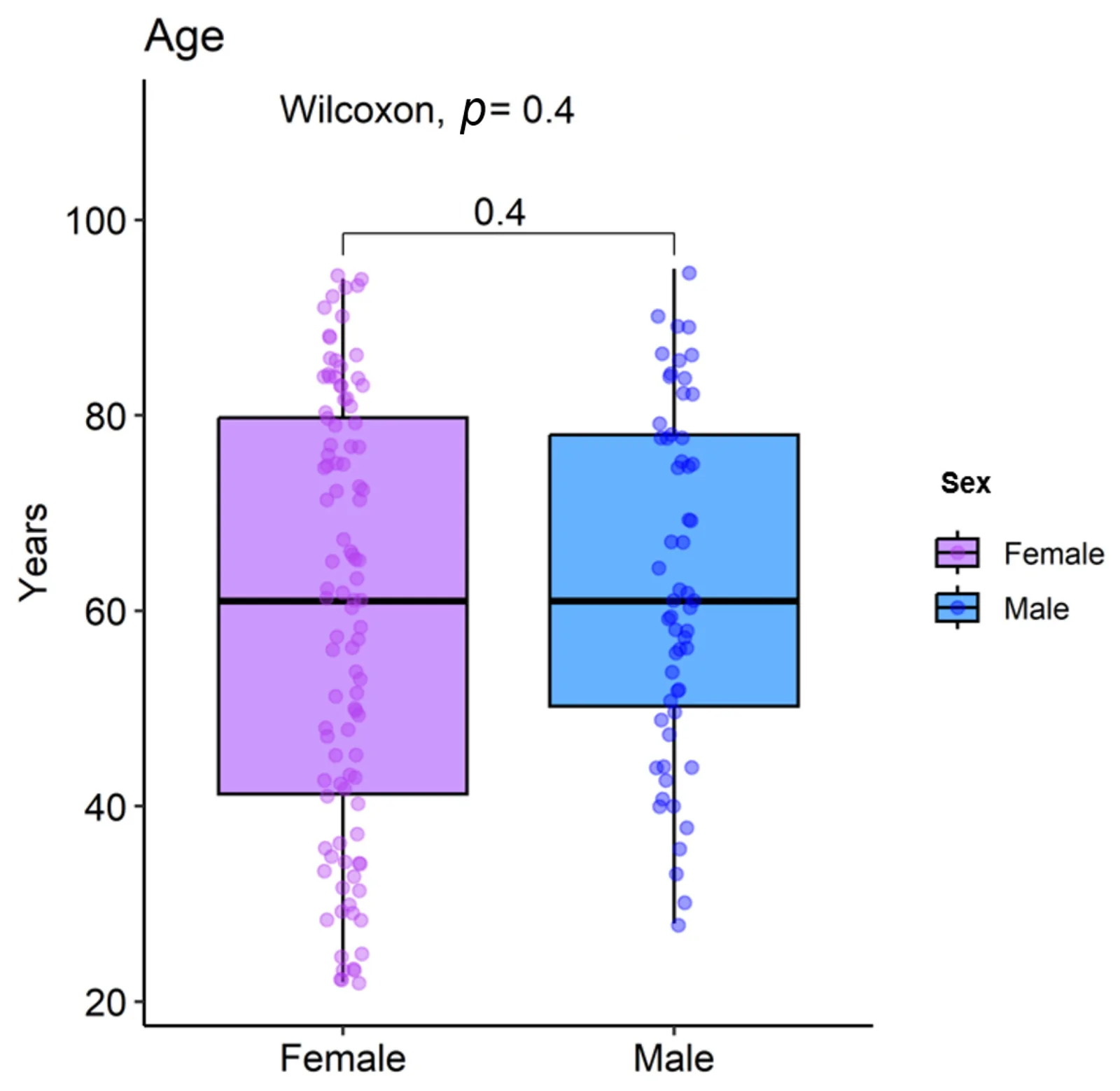
Comparison of the age of hospitalized patients by sex.

A total of 160 hospitalized patients were included. Most patients were female (64%), with a median age of 61 years (IQR: 43.5–79), with no statistically significant differences between groups according to sex (*p* = 0.4) (Table 1, Figure 1).

The most frequent comorbidities were hypertension (7.5%) and type 2 diabetes mellitus (3.1%). The distribution of hypertension and type 2 diabetes mellitus was similar between men and women (*p* = 0.5 and *p* = 0.7, respectively). In a subsample of patients receiving medication, 56% were on antiplatelet therapy and 44% on anticoagulant therapy. Regarding medical history, gastrointestinal bleeding was reported in 4.4% of the population, and gastrointestinal neoplasia in 2.5%. All cases of gastrointestinal neoplasia were observed in the female group (3.9%), with no cases reported among men (*p* = 0.3).

### 3.1. Indications for FOBT Testing and Requesting Department

The fecal occult blood test (FOBT) was primarily requested for anemia, accounting for 51% of all indications, followed by melena (26%) and hematochezia (6.9%) (Table 2).

**Figure 2:**
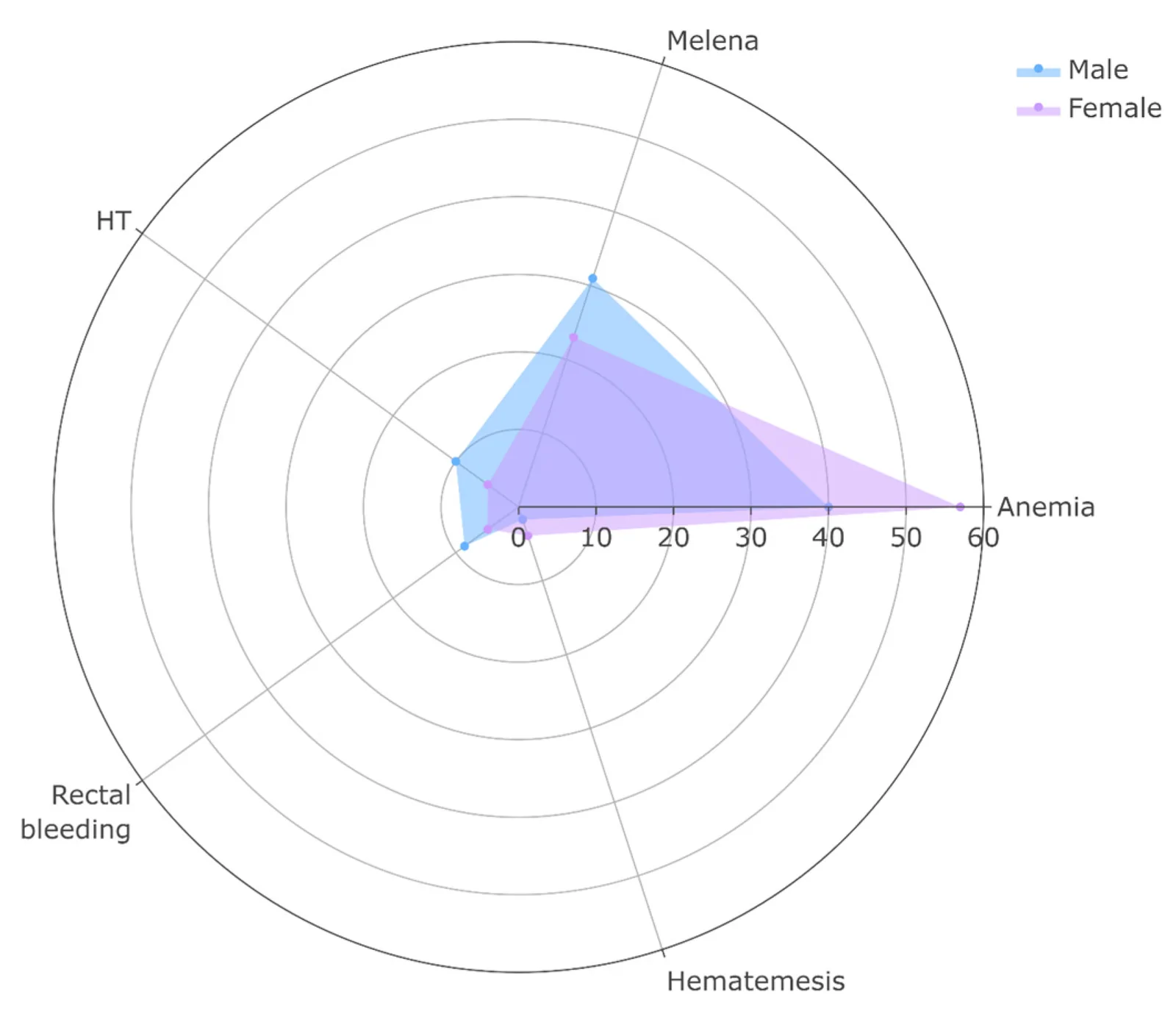
Main indications for FOBT in hospitalized patients by sex. FOBT: Fecal Occult Blood Test; HT: Hematochezia.

The indication of anemia showed a statistically significant difference between sexes (*p* = 0.036), being more frequent among female patients (57%) than male patients (40%). In contrast, indications for hematochezia (10% in men vs. 4.9% in women) and melena (31% in men vs. 23% in women) (Figure 2) were more common in men; however, these differences did not reach statistical significance (all *p* > 0.05).

**Figure 3:**
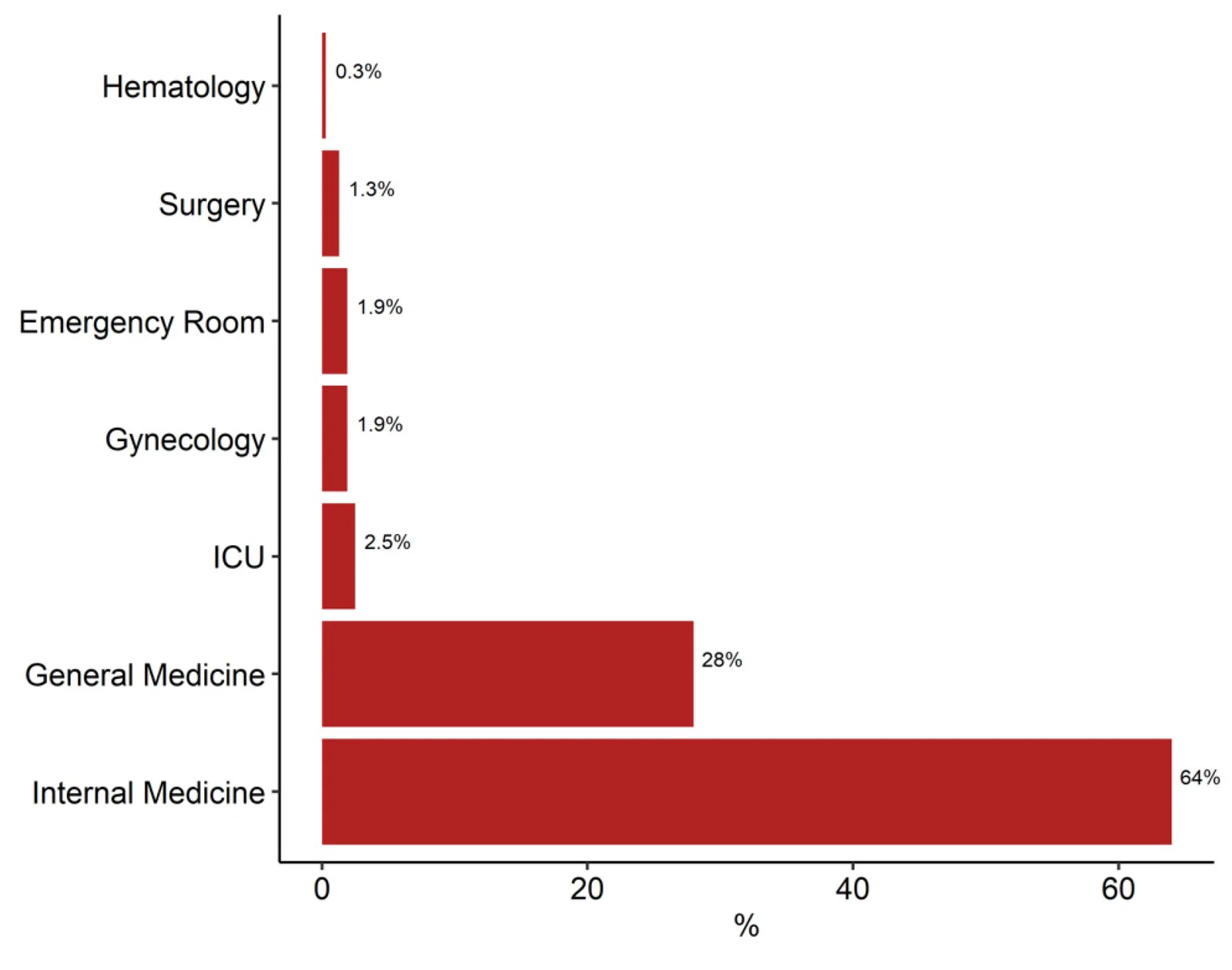
Distribution of requesting departments for FOBT in hospitalized patients. FOBT: Fecal Occult Blood Test.

The department most frequently requesting the test was Internal Medicine (64%), followed by General Medicine (28%). No statistically significant differences were observed between sexes in the departments with the highest request volume (Internal Medicine, *p* = 0.4; General Medicine, *p* = 0.09). General Medicine showed a trend toward more frequent requests among male patients (34% vs. 24% in female patients) (Figure 3). The Emergency Department, Surgery, and Hematology requested the test exclusively in female patients, each representing 2.9% or less of the total female population.

### 3.2. Admission and Reason for Consultation

Among the 160 patients, the FOBT was positive in the majority of cases (N = 98; 61.3%). Baseline characteristics such as age (median: 61 years vs. 62 years; *p* = 0.82) and sex distribution (*p* = 0.93) were similar between patients with positive and negative FOBT results (Table 3).

**Figure 4:**
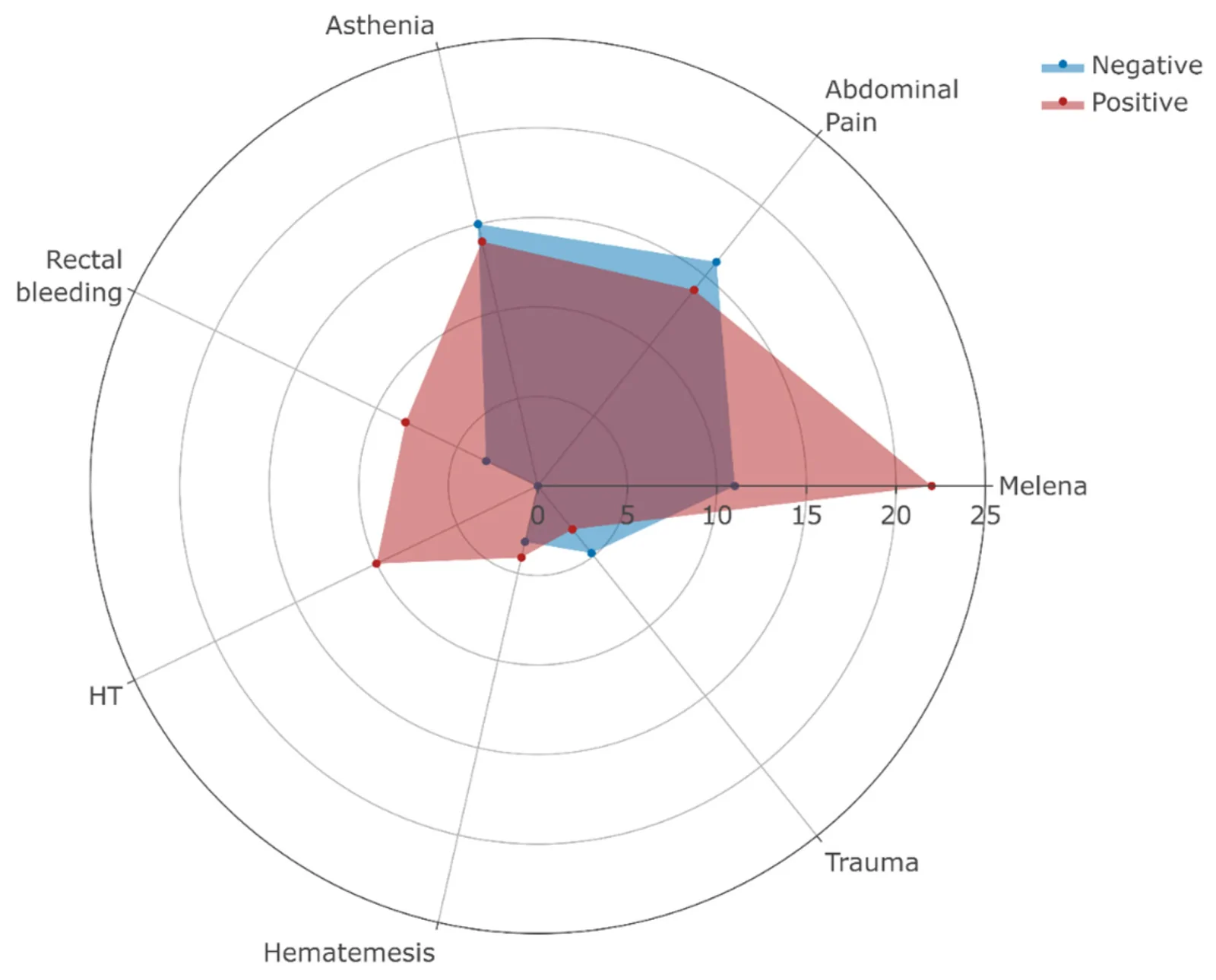
Main reasons for consultation according to FOBT results. HT: Hematochezia; FOBT: Fecal Occult Blood Test.

The admission diagnosis of anemia was significantly more frequent in the FOBT-positive group (82%) compared with the FOBT-negative group (68%) (*p* = 0.044). Regarding reasons for consultation, hematochezia was reported in 10% of patients with a positive FOBT, whereas none of the patients with a negative FOBT presented this finding (*p* = 0.007). Although not statistically significant, the presence of melena was more frequent in the FOBT-positive group (22%) than in the FOBT-negative group (11%) (*p* = 0.074). Other reasons for consultation, such as abdominal pain, asthenia, or trauma, did not show relevant differences between groups (all *p* > 0.05) (Figure 4).

### 3.3. Relationship Between FOBT, Esophagogastroduodenoscopy, and Colonoscopy

Endoscopic verification was performed unevenly according to FOBT result: 71% of FOBT-positive patients underwent EGD and/or colonoscopy, versus 29% of FOBT-negative patients. The analysis of esophagogastroduodenoscopy (EGD) did not show a statistically significant association between abnormal findings and FOBT positivity (*p* = 0.22). Among patients with a positive FOBT who underwent EGD, 64% presented abnormal findings, compared with 47% in the FOBT-negative group. Gastric/duodenal ulcer was a frequent finding, present in 23.6% of EGD-tested patients (29.1% in the FOBT-positive group vs. 5.9% in the FOBT-negative group). Among ulcers classified according to the Forrest classification, type III was the most frequent pattern (76%). Suspicion of neoplasia on EGD was higher in the FOBT-positive group (9.1% vs. 5.9%); however, this difference was not statistically significant (*p* = 1.00) (Table 4).

Regarding colonoscopy findings, the prevalence of abnormal results was higher in the FOBT-positive group (57%) compared with the FOBT-negative group (25%); however, this difference did not reach statistical significance (*p* = 0.32). When restricted to patients who actually underwent colonoscopy, no specific finding—including diverticulosis (25% vs. 34%), hemorrhoids, or suspected neoplasia—showed a statistically significant association with FOBT result (all *p* ≥ 0.19), reflecting the small verified subsample (n = 39) rather than a true absence of effect.

### 3.4. Multivariable and Bias-Corrected Analysis

In multivariable analysis, FOBT positivity was not an independent predictor of abnormal EGD (OR 2.01, 95% CI 0.66–6.10) or abnormal colonoscopy (OR 4.58, 95% CI 0.39–54.3), and neither age nor antiplatelet/anticoagulant use reached significance (Table 5).

After correcting for differential verification (Begg–Greenes method), sensitivity of FOBT decreased and specificity increased relative to naive estimates for both EGD (sensitivity 81% → 68%; specificity 31% → 48%) and colonoscopy (95% → 78%; 17% → 53%). Multiple imputation of non-verified cases yielded results consistent with the bias-corrected estimates for both outcomes. Likelihood ratios close to 1 for both outcomes indicate that FOBT result produces minimal shift in post-test probability of pathology (Table 6).

## 4. Discussion

This study evaluated the clinical utility of fecal occult blood testing (FOBT) in hospitalized patients and provides important insights into its real-world diagnostic role beyond its traditional application in colorectal cancer screening. In this cohort of 160 hospitalized adults, FOBT demonstrated a high positivity rate (61.3%) but showed no robust, independent association with endoscopic pathology, upper or lower, once differential endoscopic verification was accounted for.

Importantly, beyond its diagnostic performance, our findings highlight a persistent pattern of inappropriate FOBT utilization in hospitalized patients. A substantial proportion of tests were requested in clinical scenarios involving overt gastrointestinal bleeding, such as melena and hematochezia, where current guidelines clearly recommend direct endoscopic evaluation without prior fecal testing [8,9,10]. This reflects a gap between evidence-based recommendations and real-world practice, consistent with previous reports describing inappropriate inpatient FOBT use across different healthcare settings [5].

Anemia was the most frequent indication for FOBT, accounting for more than half of all tests performed. This observation aligns with prior studies indicating that FOBT is often incorporated into the diagnostic workup of unexplained anemia in hospitalized patients rather than being restricted to screening contexts [8,10,22]. The higher frequency of FOBT requests for anemia among female patients may reflect the increased prevalence of iron deficiency anemia in women, as well as a lower clinical threshold for diagnostic evaluation in this population [10,23].

The positivity rate observed in our cohort markedly exceeds that reported in screening populations, where rates typically range between 5% and 10% [2,24]. This difference is expected given the clinical complexity of hospitalized patients, who frequently present with anemia, suspected gastrointestinal bleeding, and multiple comorbidities. In this context, FOBT positivity was significantly associated with both the admission diagnosis of anemia and the presence of hematochezia, supporting its role as a marker of ongoing or recent gastrointestinal blood loss.

Notably, hematochezia was observed exclusively in FOBT-positive patients, further reinforcing the association between a positive test and lower gastrointestinal bleeding. This finding is consistent with prior evidence suggesting that, in symptomatic populations, FOBT positivity correlates more strongly with distal gastrointestinal sources than with upper tract pathology [9,12].

A key finding of this study was the absence of a significant association between FOBT positivity and abnormal findings on esophagogastroduodenoscopy (EGD). Clinically relevant lesions such as gastric and duodenal ulcers were identified in both FOBT-positive and FOBT-negative groups, indicating that FOBT does not reliably discriminate upper gastrointestinal sources of bleeding. This observation is in line with previous studies demonstrating the limited sensitivity of FOBT for upper gastrointestinal bleeding, largely due to hemoglobin degradation during intestinal transit [4,12].

Endoscopic verification was performed far more often in FOBT-positive than FOBT-negative patients (71% vs. 29% underwent any endoscopic evaluation), and unadjusted comparisons of specific findings such as diverticulosis initially appeared to favor the FOBT-positive group. When restricted to verified patients, however, none of these associations remained significant, and this held across complete-case, verification-bias-corrected, and multiple-imputation analyses (Section 3.4)—indicating that the crude association reflected differential verification rather than a true diagnostic signal.

From a clinical perspective, these findings do not support using FOBT positivity to prioritize endoscopic evaluation in hospitalized patients. Its principal clinically actionable signal in this cohort was institutional: frequent use in scenarios of overt bleeding where guidelines already indicate direct endoscopy, which may reduce diagnostic efficiency, delay definitive evaluation, and increase unnecessary healthcare utilization [5,6].

From an institutional standpoint, the predominance of FOBT requests by Internal Medicine reflects its central role in managing complex hospitalized patients. Nevertheless, the frequent use of FOBT in patients with overt gastrointestinal bleeding underscores persistent inappropriate use despite well-established guideline recommendations. Current international guidelines, including those from the American College of Gastroenterology and the U.S. Preventive Services Task Force, restrict FOBT use to colorectal cancer screening in asymptomatic individuals or evaluation of iron-deficiency anemia in the absence of visible bleeding [1,10,12,23,24]. Its use in patients with melena or hematochezia offers minimal diagnostic value and may contribute to delays in appropriate management.

These findings are particularly relevant in low- and middle-income settings, where inefficient use of diagnostic resources may have a disproportionate impact on healthcare systems. In such contexts, inappropriate FOBT utilization may contribute to unnecessary testing, increased costs, and delayed access to definitive procedures such as colonoscopy.

This study has several limitations. Its retrospective design may introduce selection bias and limit causal inference. Additionally, indication bias must be considered, as FOBT was ordered based on clinician judgment, potentially influencing both test utilization and subsequent diagnostic pathways. Verification bias was substantial and directly quantifiable: 71% of FOBT-positive versus 29% of FOBT-negative patients underwent endoscopic evaluation. We addressed this through complete-case analysis restricted to verified patients, Begg–Greenes correction of diagnostic accuracy estimates [20], and multiple imputation as a sensitivity analysis [18]; all three approaches converged on the absence of a significant FOBT-pathology association. Nonetheless, the verified colonoscopy subgroup was small (n = 39), yielding wide confidence intervals that cannot exclude a true association of smaller magnitude. The single-center design further limits generalizability, and the sample size may have reduced power for detecting associations with less frequent outcomes such as neoplasia.

## 5. Conclusions

FOBT demonstrated no robust, independent association with endoscopic pathology—upper or lower—once differential verification was accounted for, and it is frequently used inappropriately in scenarios where direct endoscopic evaluation is already indicated. These findings do not support using FOBT positivity to prioritize colonoscopy and instead identify guideline-discordant institutional use as the main modifiable target for improving diagnostic efficiency in inpatient care.

## Figures and Tables

**Table 1:** Baseline characteristics of hospitalized patients.

Characteristic	Overall (n = 160 ^1^)	Female (n = 102 ^1^)	Male (n = 58 ^1^)	*p*-Value
Age, years	61 [43.5–79]	61 [41–80]	61 [50–78]	0.4 ^2^
Comorbidities				
Hypertension	12 (7.5%)	9 (8.8%)	3 (5.2%)	0.5 ^3^
Type 2 diabetes mellitus	5 (3.1%)	4 (3.9%)	1 (1.7%)	0.7 ^3^
Medication				0.6 ^3^
Antiplatelet therapy	10 (56%)	6 (67%)	4 (44%)	
Anticoagulant therapy	8 (44%)	3 (33%)	5 (56%)	
Medical history				
Gastrointestinal bleeding	7 (4.4%)	3 (2.9%)	4 (6.9%)	0.3 ^3^
Colon polyp	1 (0.6%)	1 (1.0%)	0 (0%)	>0.9 ^3^
Gastrointestinal neoplasia	4 (2.5%)	4 (3.9%)	0 (0%)	0.3 ^3^

HTA: Hypertension; DM2: Type 2 Diabetes Mellitus; GI: Gastrointestinal. ^1^ Median [Q1–Q3]; n (%); ^2^ Wilcoxon rank-sum test. ^3^ Fisher’s exact test.

**Table 2:** Main indications and requesting department for FOBT in hospitalized patients by sex.

Characteristic	Overall (n = 160 ^1^)	Female (n = 102 ^1^)	Male (n = 58 ^1^)	*p*-Value
Indication				
Anemia	81 (51%)	58 (57%)	23 (40%)	0.036 ^2^
Melena	41 (26%)	23 (23%)	18 (31%)	0.2 ^2^
Hematochezia	11 (6.9%)	5 (4.9%)	6 (10%)	0.2 ^3^
Rectal bleeding	10 (6.3%)	5 (4.9%)	5 (8.6%)	0.5 ^3^
Hematemesis	5 (3.1%)	4 (3.9%)	1 (1.7%)	0.7 ^3^
Department				
Internal Medicine	103 (64%)	67 (66%)	36 (62%)	0.4 ^3^
General Medicine	44 (28%)	24 (24%)	20 (34%)	0.09 ^3^
ICU	4 (2.5%)	2 (2.0%)	2 (3.4%)	0.7 ^2^
Gynecology	3 (1.9%)	3 (2.9%)	0 (0%)	–
Emergency Department	3 (1.9%)	3 (2.9%)	0 (0%)	–
Surgery	2 (1.3%)	2 (2.0%)	0 (0%)	–
Hematology	1 (0.6%)	1 (1.0%)	0 (0%)	–

^1^ n (%); ^2^ Pearson’s chi-squared test; ^3^ Fisher’s exact test.

**Table 3:** Admission characteristics and main reasons for consultation according to FOBT positivity.

Characteristic	Overall (n = 160 ^1^)	FOBT Negative (n = 62 ^1^)	FOBT Positive (n = 98 ^1^)	*p*-Value
Age, years	61.0 [43.5–79.0]	62.0 [43.0–77.0]	61.0 [44.0–79.0]	0.8 ^2^
Sex				0.9 ^3^
Female	102 (64%)	40 (65%)	62 (63%)	
Male	58 (36%)	22 (35%)	36 (37%)	
Admission diagnosis				
Anemia	122 (76%)	42 (68%)	80 (82%)	0.044 ^3^ OR 2.12, 95% CI 1.01–4.43
Thrombocytopenia	2 (1.3%)	1 (1.6%)	1 (1.0%)	>0.9 ^4^
Reason for consultation				
Melena	29 (18%)	7 (11%)	22 (22%)	0.074 ^3^
Abdominal pain	24 (15%)	10 (16%)	14 (14%)	0.8 ^3^
Asthenia	23 (14%)	9 (15%)	14 (14%)	>0.9 ^3^
Rectal bleeding	10 (6.3%)	2 (3.2%)	8 (8.2%)	0.3 ^4^
Hematochezia	10 (6.3%)	0 (0%)	10 (10%)	0.007 ^4^ OR 14.83 95% CI 0.85–257.80 ^5^
Hematemesis	6 (3.8%)	2 (3.2%)	4 (4.1%)	>0.9 ^4^
Trauma	6 (3.8%)	3 (4.8%)	3 (3.1%)	0.7 ^4^

FOBT: fecal occult blood test; ^1^ Median [Q1–Q3]; n (%); ^2^ Wilcoxon rank-sum test; ^3^ Pearson’s chi-squared test; ^4^ Fisher’s exact test; ^5^ Odds ratio calculated with Haldane–Anscombe continuity correction due to a zero cell in the FOBT-negative group; the wide confidence interval reflects instability of this estimate, and Fisher’s exact test is considered the more reliable measure of association for this comparison.

**Table 4:** Relationship between FOBT, esophagogastroduodenoscopy, and colonoscopy in hospitalized patients.

Characteristic	Overall	FOBT Negative	FOBT Positive	*p*-Value
EGD, n	72	17	55	
Result				0.22 ^2^
Abnormal	43/72 (59.7%)	8/17 (47.1%)	35/55 (63.6%)	
Normal	29/72 (40.3%)	9/17 (52.9%)	20/55 (36.4%)	
Suspected neoplasia	6/72 (8.3%)	1/17 (5.9%)	5/55 (9.1%)	1.00 ^3^
Gastric neoplasia	2/72 (2.8%)	1/17 (5.9%)	1/55 (1.8%)	0.42 ^3^
Gastric/Duodenal ulcer	17/72 (23.6%)	1/17 (5.9%)	16/55 (29.1%)	—
Forrest classification				>0.90 ^3^
IIA	4/17 (23.5%)	0/1 (0%)	4/16 (25.0%)	
III	13/17 (76.5%)	1/1 (100%)	12/16 (75.0%)	
Esophageal varices	3/72 (4.2%)	1/17 (5.9%)	2/55 (3.6%)	0.56 ^3^
Endoscopic treatment	9/72 (12.5%)	2/17 (11.8%)	7/55 (12.7%)	1.0 ^3^
Colonoscopy, n	39	4	35	
Result				0.32 ^3^
Abnormal	21/39 (53.8%)	1/4 (25.0%)	20/35 (57.1%)	
Normal	18/39 (46.2%)	3/4 (75.0%)	15/35 (42.9%)	
Suspected neoplasia	3/39 (7.7%)	0/4 (0%)	3/35 (8.6%)	1.0 ^3^
Colorectal neoplasia	1/39 (2.6%)	0/4 (0%)	1/35 (2.9%)	
Mucinous—ileal	1/39 (2.6%)	0/4 (0%)	1/35 (2.9%)	
Hemorrhoids	4/39 (10.3%)	0/4 (0%)	4/35 (11.4%)	1.0 ^3^
Diverticulosis	13/39 (33.3%)	1/4 (25.0%)	12/35 (34.3%)	1.0 ^3^
Endoscopic treatment	2/39 (5.1%)	1/4 (25.0%)	1/35 (2.9%)	0.197 ^3^

EGD: esophagogastroduodenoscopy; FOBT: fecal occult blood test. Data are presented as n/N (%), where N corresponds to the number of patients who underwent the respective endoscopic procedure; for the Forrest classification, N corresponds to patients with gastric/duodenal ulcer. ^2^ Pearson’s chi-squared test; ^3^ Fisher’s exact test.

**Table 5:** Multivariable logistic regression for abnormal endoscopic findings.

Outcome/Covariate	OR	95% CI	*p*-Value
EGD abnormal (n = 72)			
FOBT positive	2.01	0.66–6.10	0.22
Age	1.00	0.98–1.03	0.90
Antiplatelet/anticoagulant use	1.25	0.25–6.22	0.79
Colonoscopy abnormal (n = 39) ^1^			
FOBT positive	4.58	0.39–54.3	0.23
Age	1.04	1.00–1.08	0.059

^1^ Antiplatelet/anticoagulant use was excluded from the colonoscopy model due to quasi-complete separation (only 1 of 39 verified patients was on antiplatelet/anticoagulant therapy).

**Table 6:** Diagnostic accuracy of FOBT for abnormal endoscopic findings—naive vs. verification-bias-corrected estimates.

Measure	EGD (Naive)	EGD (Corrected ^1^)	Colonoscopy (Naive)	Colonoscopy (Corrected ^1^)
Sensitivity	81.4% (67.4–90.3)	68.1% (54.9–83.0)	95.2% (77.3–99.2)	78.3% (49.7–100.0)
Specificity	31.0% (17.3–49.2)	47.9% (30.1–63.9)	16.7% (5.8–39.2)	52.5% (0.0–69.1)
PPV	63.6% (50.4–75.1)	63.6% (50.4–75.1)	57.1% (40.9–72.0)	57.1% (40.9–72.0)
NPV	52.9% (31.0–73.8)	52.9% (31.0–73.8)	75.0% (30.1–95.4)	75.0% (30.1–95.4)
Positive LR	1.18 (0.89–1.57)	1.31 (0.84–2.16)	1.14 (0.91–1.44)	1.65 (0.53–3.17)
Negative LR	0.60 (0.26–1.37)	0.66 (0.30–1.36)	0.29 (0.03–2.51)	0.41 (0.00–1.38)
Overall accuracy	61.1% (49.6–71.5)	59.5% (47.0–72.2)	59.0% (43.4–72.9)	64.0% (38.7–80.9)

LR: likelihood ratio. ^1^ Corrected for differential verification using the method of Begg and Greenes [20,21]. PPV and NPV are mathematically invariant under this correction, as they depend only on disease prevalence within each FOBT-result stratum, estimated directly from verified patients. 95% CIs for naive estimates use the Wilson score method (proportions) and standard log-normal method (likelihood ratios); 95% CIs for corrected estimates were obtained by bootstrap resampling (3000 replicates).

## Data Availability

The data supporting the findings of this study are available from the corresponding author upon reasonable request. The data are not publicly available due to patient privacy and ethical restrictions associated with the use of clinical medical records.
